# ^18^F-FDG PET Imaging in the Evaluation of Treatment Response to New Chemotherapies beyond Imatinib for Patients with Gastrointestinal Stromal Tumors

**DOI:** 10.5402/2011/824892

**Published:** 2011-08-14

**Authors:** Antonella Stefanelli, Giorgio Treglia, Paoletta Mirk, Barbara Muoio, Alessandro Giordano

**Affiliations:** ^1^Institute of Nuclear Medicine, Catholic University of the Sacred Heart, Largo Gemelli, 8, 00168 Rome, Italy; ^2^Institute of Radiology, Catholic University of the Sacred Heart, Largo Gemelli, 8, 00168 Rome, Italy; ^3^School of Medicine, Catholic University of the Sacred Heart, Largo Gemelli, 8, 00168 Rome, Italy

## Abstract

*Aim*. ^18^F-fluorodeoxyglucose positron emission tomography (^18^F-FDG PET) is a powerful tool for staging and defining “good responders” to chemotherapy in tumor setting. Gastrointestinal stromal tumors (GISTs) are sarcoma involving gastrointestinal tract and may require a chemotherapy including imatinib, a tyrosine kinase inhibitor agent. Some GIST patients become refractory to imatinib; therefore, other tyrosine kinase inhibitors or concomitant chemotherapy may be considered for treatment. The aim of this paper is to assess if ^18^F-FDG PET imaging is a useful tool to evaluate treatment response to new chemotherapies beyond imatinib for GIST patients. *Methods*. We performed a review of the literature about the role of ^18^F-FDG PET in the evaluation of treatment response to new chemotherapies beyond imatinib for GIST patients. *Results and Conclusions*. ^18^F-FDG PET seems to be able to assess therapy response earlier than computed tomography (CT) imaging in imatinib refractory GIST patients treated with other agents. However, a dual modality PET-CT imaging is recommendable to achieve a better detection of all lesions.

## 1. Introduction

Gastrointestinal stromal tumors (GISTs) are 0.1–3.0% of all cancers involving gastrointestinal tract and account for 6% of all sarcomas [1]. GISTs usually occur in middle-aged and older patients and are often asymptomatic when <5 cm in their longest dimension; GISTs become symptomatic when they grow >5 cm of diameter [2]. Patients affected by GISTs are frequently symptomatic for gastrointestinal bleeding, anemia, abdominal pain, dyspepsia, or an abdominal mass. Most common localizations of GISTs are stomach (70%), followed by the small intestine (20%), large intestine (5%), and esophagus (<5%) [3]. GISTs are benign in 70% of cases, but 30% are malignant and most common metastatic localizations occur in liver and peritoneum. Other metastatic sites include the lungs, pleura, retroperitoneum, bone, and subcutaneous tissues [1]. Prognostic factors have been proposed as tumor size <5 cm, ability to perform a complete initial resection of the tumor, and tumor grade and site; however, prediction of benign or malignant behavior of a GIST remains difficult to establish [4], for example, intestinal tumors seem to be more malignant than gastric tumors [5, 6]. Beyond the prognostic stratification, surgical resection remains the mainstay of treatment in resectable tumors, whereas recurrent and metastatic GISTs are poor responders to chemotherapy and irradiation. 

The origin of GIST cells is probably related not to smooth muscle cells but to cells of Cajal [7, 8]. In fact, both GIST cells and cells of Cajal express, on the cell surface, the receptor c-kit which is identified by CD117 [9]. C-kit is a tyrosine kinase and is activated as a ligand by a stem cell factor. A mutation of the c-kit protooncogene activates the tyrosine kinase in the absence of a stem cell factor and leads to uncontrolled cell proliferation [10].

Imatinib acts as a tyrosine kinase inhibitor of tumor cells growth and resulted in an impressive response. To date, imatinib represents the first-line therapy in advanced GIST [11]. In the last years, several trials have demonstrated the efficacy of imatinib in GIST patients by using different imaging modalities in the evaluation of treatment response such as ^18^F-FDG PET and CT. ^18^F-FDG PET has been revealed as a feasible tool for early assessment of therapy response in GIST patients treated with imatinib, compared with CT scan. However, the accuracy in detecting lesions is best achieved with CT imaging; therefore, a dual modality PET-CT evaluation is recommendable at baseline and after adequate treatment in patients with GIST [11–14].

Although imatinib improved survival of metastatic GIST patients, imatinib resistance may emerge due most commonly to evolution of secondary c-kit mutations. Therefore, new drugs have been proposed as alternative systemic therapies in GIST patients once resistance or intolerance to imatinib appears, and the efficacy of these agents have been evaluated by the well-established imaging techniques like ^18^F-FDG PET and CT.

Aim of this paper is to review literature data regarding the role of ^18^F-FDG PET in evaluating treatment response to other concomitant agents or c-kit inhibitors beyond the well-known imatinib.

## 2. GIST and Other Chemotherapies beyond Imatinib: The Role of ^18^F-FDG PET Imaging in Assessing Treatment Response

Maurel et al. investigated the role of ^18^F-FDG PET and/or CT in the assessment of tumor response in GIST patients who were refractory to high-dose imatinib treatment and designed a phase 1-2 trial of doxorubicin plus imatinib. Low-dose chemotherapy combination showed promising activities, whereas PET and CT correlate poorly in this study. However, in this study, evaluation of response by PET seems to translate the efficacy better than RECIST criteria [15], and PET criteria (EORTC criteria [16]) correlate well with progression-free survival [17].

Fuster et al. also evaluated the role of ^18^F-FDG PET in assessing response to doxorubicin plus imatinib in GIST patients refractory to high dose imatinib. Of 21 patients, 6 had partial response, 9 showed stable disease, and 6 experienced progression of disease according to EORTC criteria [16] after 2 months of treatment. Poor concordance between ^18^F-FDG PET and CT was found. Moreover, a correlation was demonstrated between PET response and progression-free survival [18].

Demetri et al. designed an open-label study in GIST patients resistant or intolerant to imatinib who received sunitinib, an oral, multitargeted tyrosine kinase inhibitor. Clinical benefit was observed in 52 of the 97 patients, and a decrease in tumor glycolytic activity was shown within 7 days of starting sunitinib. For 67 patients (69%), ^18^F-FDG PET imaging data were available from baseline to at least one post-baseline scan. In 60 patients, PET imaging was performed after 1 week of sunitinib treatment and partial metabolic responses to sunitinib were detected in 43 patients, as evidenced by a decline in maximum standardized uptake value (SUVmax) of ≥25% relative to baseline. Early changes in tumor metabolism correlated with improved clinical outcome: most of the 43 patients with partial metabolic responses subsequently showed clinical benefit based on CT or magnetic resonance imaging scans. However, objective responses on CT took much longer to detect and evolved over several months, similar to the pattern observed in studies of imatinib-treated GIST patients [19].

Sunitinib was also tested by Prior et al. in 23 GIST patients who experienced imatinib therapy failure and then received one to 12 cycles of sunitinib therapy. Sunitinib was taken orally for 4 weeks once a day, followed by a 2-week-break to provide a total cycle length of 6 weeks. ^18^F-FDG PET was performed at baseline and after the first 4 weeks of sunitinib administration. Contrast-enhanced CT was performed at the end of each cycles. After 4 weeks of treatment, ^18^F-FDG PET revealed a partial metabolic response in 12 patients (52%), metabolically stable disease in seven patients (31%), and metabolically progressive disease in four patients (17%). Overall, 83% exhibited metabolic tumor control. Disease progression was subsequently demonstrated by RECIST criteria [15] in all patients with progressive disease seen on PET. These data suggested that early ^18^F-FDG PET performed after the first cycle of sunitinib treatment start may allow the assessment of therapy response. However, this study had some limitations like the small number of patients evaluated [20].

Le Cesne et al. assessed the safety and efficacy of a first-line therapy with masitinib, another tyrosine kinase inhibitor, in 30 GIST patients. Metabolic response was evaluable for 17/30 patients but 3 of these had a negative PET at baseline. After 2 months of treatment, 3/14 had a complete metabolic response, 9/14 had partial metabolic response, and 2/14 had a stable metabolic disease. Collectively, metabolic response using ^18^F-FDG PET was seen in 86% of patients, whereas masitinib induced tumor response in 20% of evaluable patients according to RECIST criteria [15] on CT images. As already reported, RECIST was not optimal compared to EORTC criteria [16] for an early response assessment of c-kit inhibitors in GIST patients since the pattern of radiological response had no prognostic value for further outcomes [21].

Benjamin et al. evaluated the role of motesanib, another c-kit inhibitor, in advanced GIST patients who failed imatinib treatment. Motesanib was taken 125 mg orally once a day for 48 weeks or until acceptable toxicity occured. Tumor size and density were evaluated by CT at baseline and after 8, 16, 24, 32, 40, and 48 weeks, whereas metabolic tumor activity was assessed by ^18^F-FDG PET at baseline and 8 weeks after therapy start. Tumor response was defined using different methods including RECIST [15] and Choi criteria [22] for CT images and EORTC criteria for PET [16]. EORTC criteria and Choi criteria detected higher objective response rates: these were 30% (in 91 evaluable cases) and 41% (in 102 evaluable cases), respectively. In this study, Choi criteria seemed to be more sensitive in identifying PET responders than RECIST criteria, since the latter detected only 3% of response rate. However, compared to the current approved sunitinib, the insufficient overall efficacy did not support further development of motesanib in GIST patients [23].

Lastly, Lassau et al. investigated the efficacy of masatinib therapy (7,5 mg/kg daily by oral route) in metastatic GIST patients by using several imaging techniques like dynamic contrast-enhanced ultrasonography, CT, and ^18^F-FDG PET imaging. CT scan was performed before treatment start and 2, 4, 6 weeks, and then every 3 months after treatment start; ^18^F-FDG PET was performed at baseline and 1 month after treatment start. Of the 20 patients included in this study, ^18^F-FDG PET imaging was available for 14. After 1 month, PET scan remained positive in 11/14 (78.6%): no change in standard uptake values (SUV) was recorded at 1 month in 4 patients, whereas an important reduction in SUV was recorded in the remaining 7 patients. These data confirmed that ^18^F-FDG PET imaging seems to be the gold standard for early assessment of tumor response not only to imatinib but also to other c-kit inhibitors, as masatinib, in GIST patients [24].

## 3. Conclusion

Intolerance or resistance to imatinib may occur in GIST patients after treatment start and often progression of the disease is observed once the therapy is stopped. For these reasons, in the last years other new c-kit inhibitors or different concomitant treatments are emerging as an alternative chemotherapy in GIST patients with advanced disease.

^18^F-FDG PET has become the gold standard for early assessment of tumor response to imatinib and seemed to have the same role in evaluating other c-kit inhibitors response. CT has more accuracy in detecting lesions but morphological imaging alone may reveal therapy response several months after treatment start. For all these reasons, a dual modality PET-CT scan is recommended in staging and re-evaluating GIST patients who undergo c-kit inhibitors therapy as well as imatinib.

On the other hand, fewer data are available from literature and further clinical trials are needed to establish the efficacy of these new chemotherapies in GIST patients by using ^18^F-FDG PET imaging.
